# Toxic tau oligomer formation blocked by capping of cysteine residues with 1,2-dihydroxybenzene groups

**DOI:** 10.1038/ncomms10216

**Published:** 2015-12-16

**Authors:** Yoshiyuki Soeda, Misato Yoshikawa, Osborne F. X. Almeida, Akio Sumioka, Sumihiro Maeda, Hiroyuki Osada, Yasumitsu Kondoh, Akiko Saito, Tomohiro Miyasaka, Tetsuya Kimura, Masaaki Suzuki, Hiroko Koyama, Yuji Yoshiike, Hachiro Sugimoto, Yasuo Ihara, Akihiko Takashima

**Affiliations:** 1 Department of Aging Neurobiology, National Center for Geriatrics and Gerontology, Obu, Aichi 474-8511, Japan; 2 Department of Stress Neurology and Neurogenesis, Max Planck Institute of Psychiatry, Kraepelinstrasse, 2-10, Munich 80804, Germany; 3 Gladstone Institute of Neurological Disease, University of California, San Francisco, California 94158-2261, USA; 4 Chemical Biology Research Group, RIKEN Center for Sustainable Resource Science (CSRS), RIKEN, Wako, Saitama 351-0198, Japan; 5 Antibiotics Laboratory, Advanced Science Institute, RIKEN, Wako, Saitama 351-0198, Japan; 6 Graduate School of Engineering, Osaka Electro-communication University (OECU), 18-8 Hatsu-cho, Osaka 572-8530, Japan; 7 Department of Neuropathology, Faculty of Life and Medical Sciences, Doshisha University, Kyotanabe, Kyoto 610-0394, Japan; 8 Department of Clinical and Experimental Neuroimaging, Center for Development of Advanced Medicine for Dementia, National Center for Geriatrics and Gerontology, Obu, Aichi 474-8511, Japan; 9 Division of Regeneration and Advanced Medical Science, Gifu University Graduate School of Medicine, Gifu 501-1194, Japan; 10 Alzheimer's Disease Project Team, National Center for Geriatrics and Gerontology, Obu, Aichi 474-8511, Japan; 11 Laboratory of Structural Neuropathology, Graduate School of Brain Science, Doshisha University, Kizugawa, Kyoto 619-0225, Japan; 12 Laboratory of Cognition and Aging, Doshisha University, Kizugawa 619-0225, Japan

## Abstract

Neurofibrillary tangles, composed of hyperphosphorylated tau fibrils, are a pathological hallmark of Alzheimer's disease; the neurofibrillary tangle load correlates strongly with clinical progression of the disease. A growing body of evidence indicates that tau oligomer formation precedes the appearance of neurofibrillary tangles and contributes to neuronal loss. Here we show that tau oligomer formation can be inhibited by compounds whose chemical backbone includes 1,2-dihydroxybenzene. Specifically, we demonstrate that 1,2-dihydroxybenzene-containing compounds bind to and cap cysteine residues of tau and prevent its aggregation by hindering interactions between tau molecules. Further, we show that orally administered DL-isoproterenol, an adrenergic receptor agonist whose skeleton includes 1,2-dihydroxybenzene and which penetrates the brain, reduces the levels of detergent-insoluble tau, neuronal loss and reverses neurofibrillary tangle-associated brain dysfunction. Thus, compounds that target the cysteine residues of tau may prove useful in halting the progression of Alzheimer's disease and other tauopathies.

## Abstract (web summary)

Aggregation of microtubule associated protein tau is one of cause of neuronal loss in tauopathies including Alzheimer's disease. Here, the authors show that compounds with a 1,2-dihydroxybenzene skeleton can modify cysteine residues in tau and block toxic tau aggregation.

## Graphical abstract

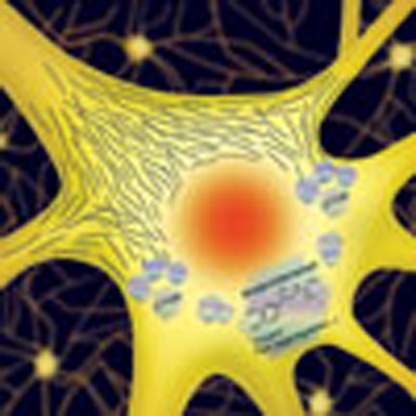

Alzheimer's disease (AD) is a progressive neurodegenerative disease, initially characterized by impaired episodic memory and eventually, severe cognitive decline. Since age is the most important risk factor for AD development, the present increase in lifespan across all demographics prioritizes the search for ameliorative and preventative treatments for the disease. Currently, cholinesterase inhibitors and *N*-methyl-D-aspartate receptor antagonists are used to treat AD symptoms with limited success; the current consensus view is that stopping disease progression will require the development of disease-modifying therapies based on defined pathogenic mechanisms^1^.

Deposition of amyloid β (Aβ) peptide in the extracellular space and formation of senile plaques, as well as the intracellular accumulation of tau protein that gives rise to neurofibrillary tangles (NFTs), with contemporaneous neuronal loss are the key pathological hallmarks of AD^2^. The amyloid hypothesis of AD posits that Aβ is the primary cause of dementia owing to its ability to induce the formation of NFT and synaptic and neuronal loss in the neocortex^3^,^4^,^5^,^6^,^7^. Tau tangle formation occurs downstream from Aβ deposition and appears to be essential for the establishment of AD; the latter view is supported by studies showing that tau depletion prevents Aβ-induced memory impairment in various lines of human amyloid precursor protein-overexpressing mice^8^,^9^.

To date, Aβ-targeted therapies (for example, immunotherapy) have failed for a variety of reasons, including failure to hamper neurodegeneration and cognitive dysfunction in patients with AD^10^,^11^. The focus of AD drug discovery research has recently shifted towards tau^12^ because, in contrast to Aβ load, tau pathology correlates with the degree of cognitive impairment^13^,^14^ and neuronal loss^15^,^16^. Tau is an attractive target because patients with frontotemporal dementia and Parkinsonism-linked to chromosome-17 (FTDP-17) carry a mutation in the tau gene and display NFT and neuronal loss in the absence of Aβ deposition^17^,^18^,^19^. Moreover, mice overexpressing mutant tau exhibit NFTs, neuronal loss and behavioural abnormalities^20^,^21^,^22^,^23^,^24^. Several lines of evidence, based on the results of experiments involving expression of the human P301L transgene in mice^25^,^26^ or the human FTDP17 mutation in fruit flies^27^, link neuronal death to the tau aggregation process, rather than to NFT formation.

Tau protein in NFTs is highly phosphorylayed, reflecting an imbalance in the activities of various kinases and phosphatases^28^,^29^. Although the role of phosphorylation in the Tau aggregation process is still debated (for example, *in vitro* evidence suggests Tau phosphorylation inhibits^30^ or has no role on tau aggregation^31^), hyperphosphorylated and/or mutated tau is suggested to adopt an alternative structure that promotes interactions between individual tau molecules. For example, our own *in vitro* experiments have proposed that tau aggregation occurs in a step-wise manner: initially, tau molecules bind to each other, through disulfide binding of their Cys residues^32^, to form soluble tau oligomers^32^,^33^; in a second step, these oligomers, comprised of ∼40 tau molecules, grow and precipitate as granular tau oligomers with a β-sheet structure; last, the granular tau oligomers bind to each other and form tau fibrils^33^.

Granular tau oligomers are detectable in the prefrontal cortex at Braak stage I, whereas NFT appear much later (Braak stage V)^34^, indicating that their formation represents a crucial early pathogenic event. Observations that neuronal death is strongly associated with the presence of Sarkosyl-insoluble tau^26^, imply that granular tau oligomers with a β-sheet structure are a major toxic species of tau and that prevention of their formation could be a promising therapeutic strategy^35^. Following this rationale, we screened a small-molecule library for compounds with the potential to inhibit the formation of granular tau oligomers. We report here that compounds containing 1,2-dihydroxybenzene inhibit granular tau oligomer formation by modifying the Cys residues of tau, thereby reducing Sarkosyl-insoluble tau levels, neuronal death and brain dysfunction in P301L tau-transgenic mice.

## Results

### Chemical array screening for tau aggregation inhibitors

**Figure 1:**
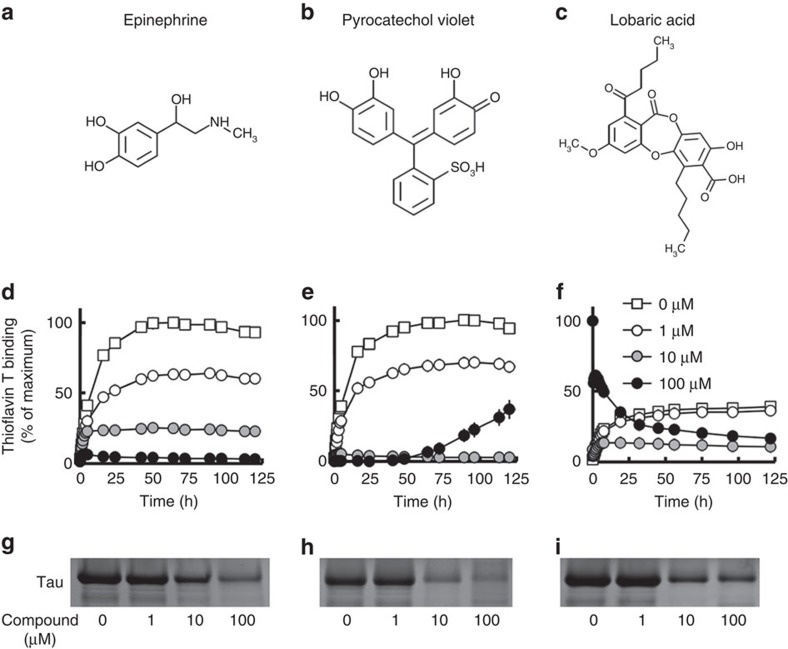
Identification of tau aggregation inhibitor. Epinephrine (**a**,**d**,**g**), pyrocatechol violet (**b**,**e**,**h**) and lobaric acid (**c**,**f**,**i**) were screened as tau aggregation inhibitors. Inhibitory effects of tau aggregation were determined by fluorescence of thioflavin T (**d**,**e**,**f**) and pelleting assay (**g**,**h**,**i**) of heparin-induced tau polymerization incubated with various concentrations of compounds (1, 10 and 100 μM). Dimethyl sulfoxide was used as the vehicle. Thioflavin T fluorescence was measured at the indicated time, and results were represented as percentage of maximum thioflavin T fluorescence (**d**,**e**,**f**;mean±s.d. of triplicate experiments; *n*=3).

To find an inhibitor of granular tau oligomer formation, we screened a series of tau-binding compounds, using a small-molecule array consisting of 6,788 compounds in the RIKEN Natural Products Depository (NPDepo). This initial screen led to the identification of 86 compounds displayed the potential to associate with tau. These compounds were subsequently assayed for thioflavin T (ThT) binding, with the exclusion of false positives using a pelleting assay in which tau was quantified in pellets derived from ultracentrifugation of a mixture of tau aggregates. Three compounds, epinephrine (Fig. 1a), pyrocatechol violet (Fig. 1b) and lobaric acid (Fig. 1c), markedly decreased ThT binding (Fig. 1d–f) and insoluble (aggregated) tau in the pellet fraction (Fig. 1g–i). As the chemical backbones of epinephrine and pyrocathechol violet consist of 1,2-dihydroxybenzene (Fig. 1a,b), we hypothesized that 1,2-dihydroxybenzene endows these compounds with the ability to inhibit tau aggregation.

### Inhibition of tau aggregation by 1,2-dihydroxybenzene

**Figure 2:**
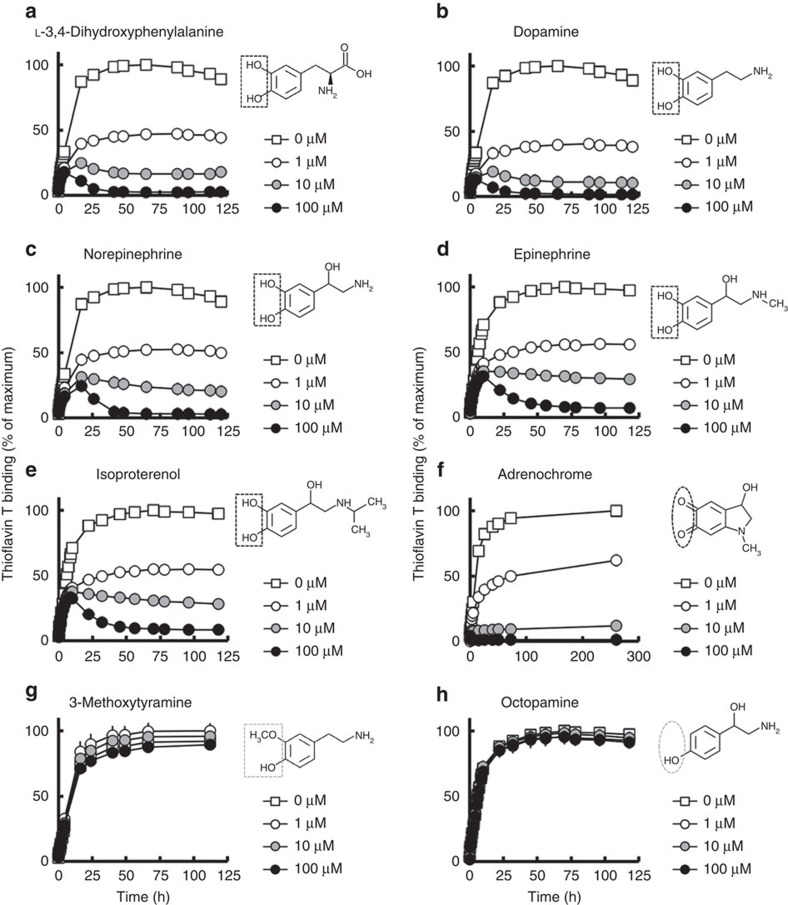
Inhibitory effects of 1,2-dihydroxybenzene-containing compounds and their derivative compounds on tau aggregation. Thioflavin T-binding activity was dose-dependently inhibited by L-3,4-dihydroxyphenylalanine (**a**), dopamine (**b**), norepinephrine (**c**), epinephrine (**d**) and isoproterenol (**e**) (1,2-dihydroxybenezene; black dotted square), and adrenochrome (**f**) (o-quinone; black dotted circle), but not by octopamine (**h**) (1-hydroxybenzene; grey dotted circle) or 3-methoxythylamine (**g**) (1-hydroxy-2-methoxybenzene; grey dotted square). Results are shown as percentage of maximum fluorescence (mean±s.d. of triplicate experiments; *n*=3).

**Figure 3:**
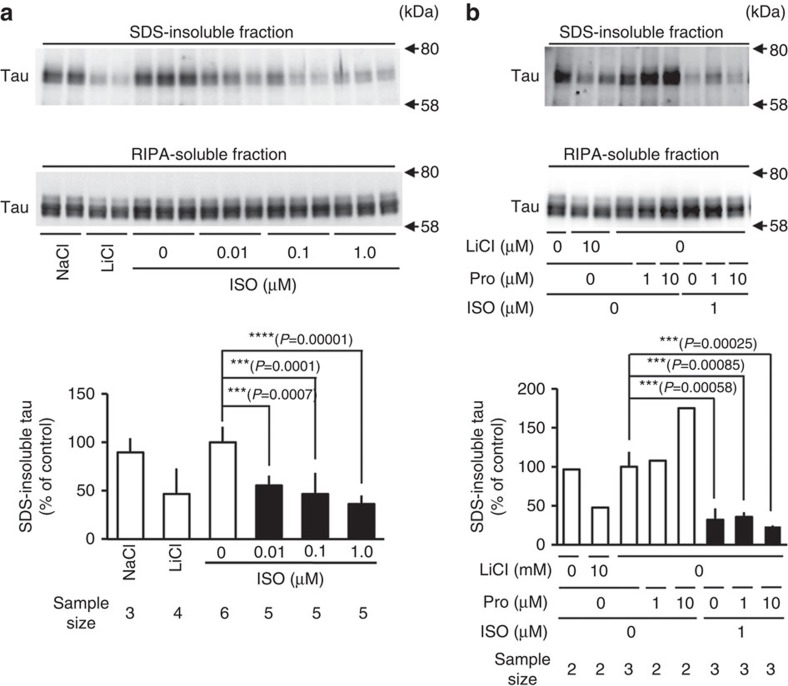
Isoproterenol inhibits tau aggregation independent of β adrenergic stimulation in cultured cells. Neuro-2a cells expressing human 2N4R tau (P301L) were treated with 0 (milliQ water), 0.01, 0.1 and 1 μM isoproterenol, 10 mM lithium chloride as a positive control as an inhibitor of glycogen synthase kinase and 10 mM sodium chloride as a negative control for 48 h. SDS-insoluble (**a**, upper part of panel) and RIPA-soluble fractions (**a**, middle part of panel) were obtained from the cell homogenates and were subjected to immunoblot analysis with JM antibody that recognized total tau. Immunoreactivity was quantified (**a**, lower part of panel), and levels of SDS-insoluble tau were normalized by corresponding RIPA-soluble tau. Results are represented as percentage of control (mean±s.d. of 3–6 experiments; 10 mM sodium chloride (*n*=3), 10 mM lithium chloride (*n*=4), vehicle (*n*=6), 0.01 μM isoproterenol (*n*=5), 0.1 μM isoproterenol (*n*=5), 1 μM isoproterenol (*n*=5)). ***P<0.001; ****P<0.0001 (one-way analysis of variance (ANOVA), with Tukey's multiple comparisons test). (**b**) Neuro-2a cells expressing human 2N4R tau (P301L) were pretreated with 0, 1 and 10 μM propranolol 30 min before treatment with 0 (milliQ water), 1 μM isoproterenol, 10 mM lithium chloride and 10 mM sodium chloride for 48 h. SDS-insoluble (**b**, upper part of panel) and RIPA-soluble (**b**, middle part of panel) fractions were obtained and subjected to immunoblot using JM antibody. Immunoreactivity was quantified (**b**, lower part of panel) and levels of SDS-insoluble tau were normalized to corresponding RIPA-soluble tau. Results are represented as percentage of control (mean±s.d. of 2–3 experiments; 10 mM sodium chloride (*n*=2), 10 mM lithium chloride (*n*=2), vehicle (*n*=3), 1 μM propranolol (*n*=2), 10 μM propranolol (*n*=2), 1 μM isoproterenol (*n*=3), 1 μM propranolol/1 μM isoproterenol (*n*=3), 10 μM propranolol/1 μM isoproterenol (*n*=3)). ***P<0.001 (one-way ANOVA, with Tukey's multiple comparisons test). ISO, isoproterenol; Pro, propranolol.

Supporting our hypothesis, we observed dose-dependent reductions in heparin-induced ThT fluorescence when cell-free preparations containing recombinant wild-type 2N4R tau were exposed to a series of 1,2-dihydroxybenzene-containing compounds. Results are shown for L-3,4-dihydroxyphenylalanine (Fig. 2a), dopamine (Fig. 2b), norepinephrine (Fig. 2c), epinephrine (Fig. 2d) and isoproterenol (ISO; Fig. 2e); adrenochrome, an oxidized form of epinephrine (Fig. 2f), also reduced ThT fluorescence. Octopamine, a compound with a 1-dihydroxybenzene structure, and 3-methoxytyramine, a catechol-O-methyltransferase-mediated metabolite of dopamine, did not alter ThT fluorescence (Fig. 2g,h), verifying the specificity of the effects produced by the compounds with a 1,2-dihydroxybenzene skeleton. This set of data showed that compounds with a 1,2-dihydroxybenzene skeleton can inhibit tau aggregation independently of their oxidation state. Subsequent investigations focused on ISO, a β1/2 adrenergic receptor agonist that can cross the blood–brain barrier (BBB)^36^,^37^; ISO is indicated for a number of medical conditions and neither interferes with neurotransmission nor induces major adverse effects. ISO treatment of Neuro2A cells engineered to stably express P301L tau^38^ results in dose-dependent reductions of SDS-insoluble tau levels in the absence of alterations in the levels of soluble tau (Fig. 3a). Importantly, the inhibitory actions of ISO on tau aggregation were not blocked by pretreatment of P301L tau-expressing Neuro2A cells with propranolol (1–10 μM), a competitive antagonist of the β adrenergic receptor (Fig. 3b), indicating that the inhibitory action of ISO on tau aggregation is not mediated through β adrenergic receptors.

**Figure 4:**
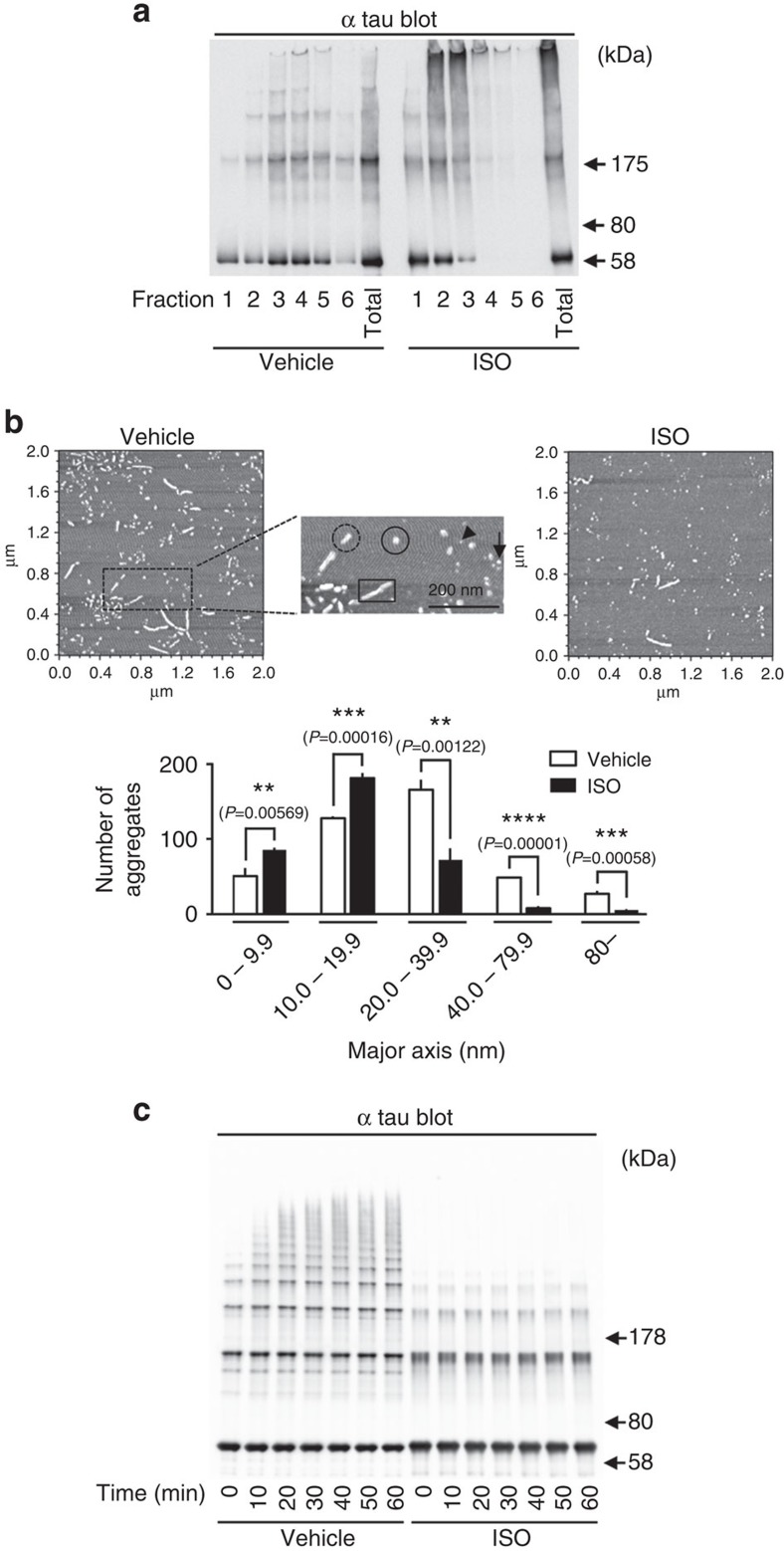
Inhibition of tau oligomer formation by isoproterenol. Heparin-induced tau aggregation mixture (120 h incubation) was subjected to sucrose density gradient centrifugation (**a**) and AFM observation (**b**). Tau aggregation mixture was separated into six fractions, and levels of tau in each fraction were analysed by western blot using tau5 antibody that recognized total tau. In AFM observation, three images were obtained from different areas (2 × 2 μm^2^) of the mica and the number and size of tau aggregates was quantitated using the image analysis programme, Matlab. Sizes of tau aggregates are 0–9.9 nm (arrowhead), 10.0–19.9 nm (arrow), 20.0–39.9 nm (black solid circle), 40.0–79.9 nm (black dotted circle) and not less than 80 nm (black solid square). Results represented as mean±s.d. of three experiments (*n*=3; **b**, lower panel). **P<0.01; ***P<0.001; ****P<0.0001 (unpaired Student's *t*-test). In first 60 min of heparin-induced tau aggregation, higher order tau oligomer was formed in soluble fraction with longer incubation time (**c**, vehicle), but growth of tau oligomer was not seen in the presence of isoproterenol (**c**, ISO). Tau oligomer in soluble fraction was detected by non-reducing condition using tau5 antibody.

We subsequently made a detailed examination of heparin-induced tau aggregates to gain further insight into the mechanism of action of ISO. Sucrose density gradient centrifugation demonstrated the absence of filamentous tau in ISO-treated (100 μM) tau aggregate mixtures (fractions 4–6; Fig. 4a). This analysis also showed that ISO markedly reduced granular tau oligomer levels (fraction 3) while increasing the levels of tau in fractions 1–2 (Fig. 4a). Analysis of heparin-induced tau aggregates by atomic force microscopy (AFM) revealed that ISO treatment leads to a significant reduction of the number of tau filaments and granular tau oligomers and an increase in the number of small tau granules (Fig. 4b). These observations suggest that ISO maintains tau in a soluble form or limits its conversion to small, amorphous granules. This interpretation is consistent with our finding that, under non-reducing conditions, ISO prevents tau molecules from forming soluble oligomers (Fig. 4c).

### Inhibitory mechanism of 1,2-dihydroxybenzene

**Figure 5:**
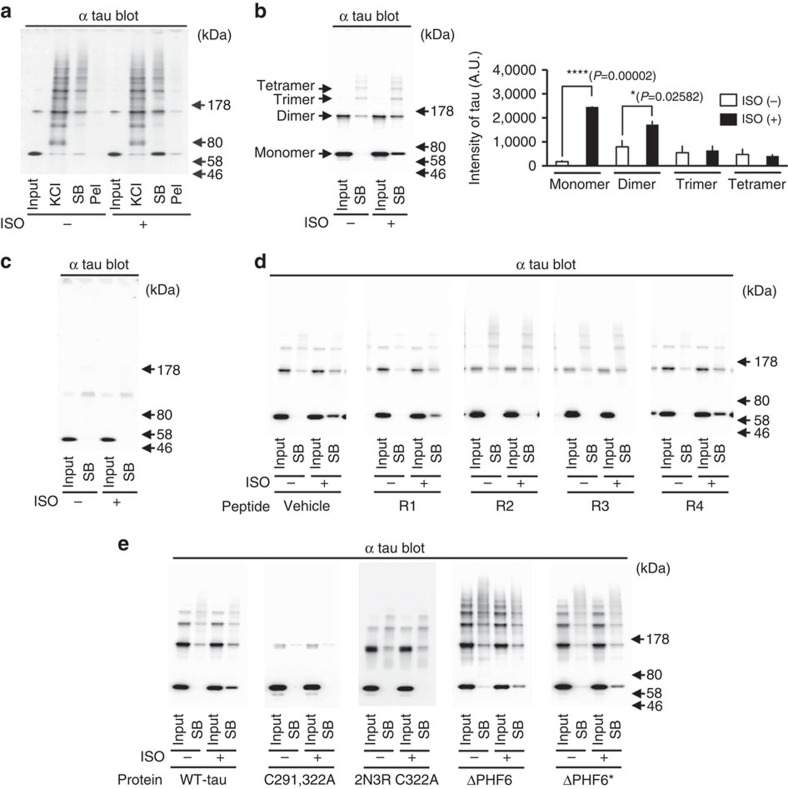
Identification of isoproterenol (ISO)-binding site on tau. ISO and tau binding were performed according to the protocol described in Supplementary Fig. 1 and ISO-bound tau was recovered in KCl, SB and Pel. Each fraction was subjected to immunoblot analysis with tau5 antibody (**a**). In SB fraction, tau monomer and oligomer were detected from an eluate of FG beads with ISO (ISO (+)) and without ISO (ISO (−); **b**, left part of panel). Levels of tau monomer and oligomer were quantified (**b**, right part of panel) and shown as mean±s.d. (triplicate experiments; *n*=3). *P<0.05; ****P<0.0001 (unpaired Student's *t*-test). FG beads with ISO (ISO (+)) and without ISO (ISO (−)) were reacted with ΔMTBR-tau. Tau was not recovered in SB fraction (**c**). Pretreated ISO with R1 (tapvpmpdlknvkskigstenlkhqpgggk), R2 (vqiinkkldlsnvqskcgskdnikhvpgggs), R3 (vqivykpvdlskvtskcgslgnihhkpgggq) and R4 (vevksekldfkdrvqskigsldnithvpgggn) peptides was incubated with wild-type (WT) 2N4R tau. Pretreated IS0 with R2 and R3 could not bind to tau, but R1 and R4 pretreatment could bind (**d**). C291, 322A-2N4R tau and C322A-2N3R tau did not show tau band as well as tau incubating beads without ISO, ISO (+), but WT 2N4R tau, ΔPHF6-tau and ΔPHF6*-tau showed tau band as ISO-bound tau (**e**). MTBR, microtubule-binding domain repeat.

ISO-coated magnetic FG (Functional magnetic) beads (Supplementary Fig. 1) were used to identify the binding site of 1,2-dihydroxybenzene compounds to tau; for this, FG beads were incubated with tau in the presence (+) or absence (−) of ISO before elution with either 1 M KCl or sample buffer (SB). As shown in Fig. 5a, tau-ISO complexes could be efficiently ut not KCl, indicating that the protein–drug interaction depends on non-ionic bonding. Further, we observed that ISO bound monomeric, dimeric, trimeric and tetrameric forms of tau. Compared with when ISO was not included in the incubation mixture, FG beads bound monomeric tau more strongly (14.1-fold) than dimeric tau (2.2-fold) in the presence of ISO, whereas the amounts of trimeric and tetrameric tau bonding to the FG beads were not influenced by ISO (Fig. 5b). Thus, these results demonstrate that ISO primarily associates with monomeric species of tau.

The site at which ISO binds to tau was further explored by comparing the binding of the drug to wild-type tau and a mutant form of tau that lacks the microtubule-binding region (ΔMTBR). As tau could only be eluted (recovered) when wild-type tau was used (Fig. 5c), we conclude that ISO binding to tau depends on the presence of MTBR. More precise information regarding the ISO-MTBR site of interaction was obtained by pre-treating FG beads with peptides corresponding to each of the four repeats of tau (R1–R4) before the addition of the drug and subsequent recovery of ISO-tau complexes. Such recovery was only possible from beads that had been pre-treated with vehicle or R1 and R4 peptides (Fig. 5d), indicating that ISO binds to sites localized in the R2 and/or R3 regions of tau.

**Figure 6:**
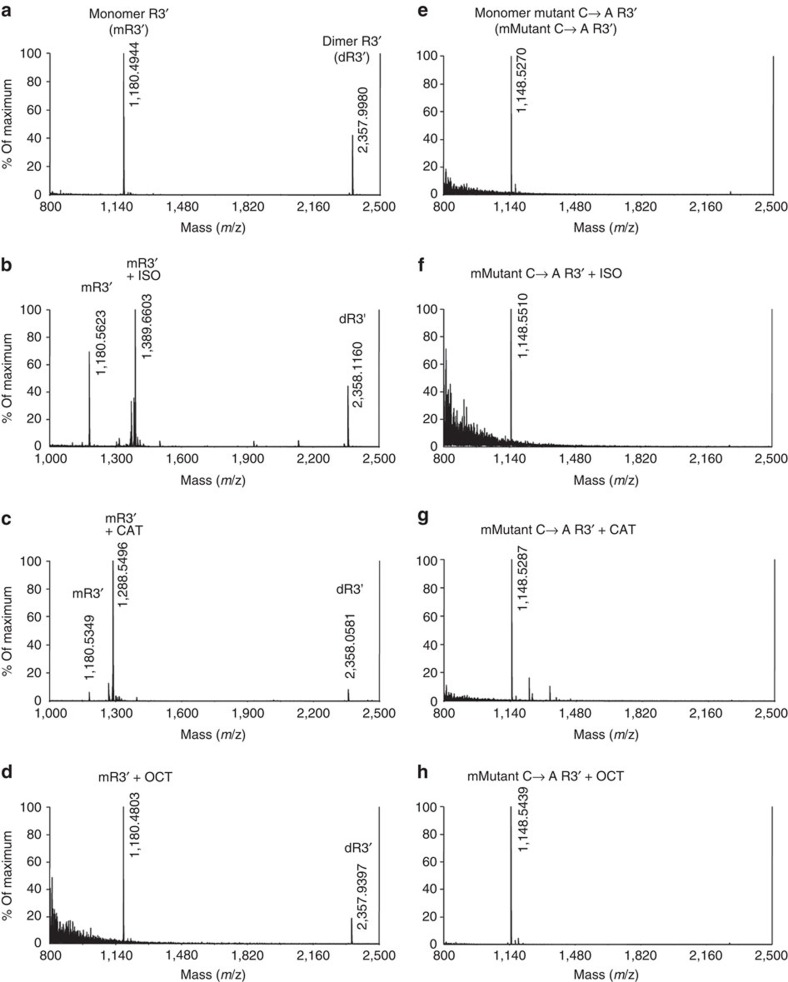
Binding of 1,2-dihydroxybenzene-containing compounds with Cys residue of tau by mass spectrometry analysis. Vehicle (milliQ water; **a**) and 1-hydroxybenzene-containing octopamine (OCT; MW=153.2; **d**) were used as negative controls. Compounds containing 1,2-dihydroxybenzene isoproterenol (ISO; MW=211.3; **b**) and pyrocatechol (CAT; MW=110.1; **c**) incubated with tau R3 partial peptide, R3' (skvtskcgslgn; MW=1,180.3) showed ISO/R3' and CAT/R3 signal in mass spectrum (**b**,**c**). Mutant C→A R3' peptide (skvtskagslgn; MW=1,148.2; **e**–**h**) incubated with vehicle (**e**), ISO (**f**), CAT (**g**) and OCT (**h**) only showed mutant R3' signal. Data are normalized to the each maximum mass spectrometry signal.

The R2 and R3 regions of tau are characterized by cysteine residues (positions 291 and 322) and hexapeptides (positions 275–280 in PHF6*; positions 306–311 in PHF6); these regions are critical for tau fibril formation^39^,^40^,^41^. By testing the ability of ISO to bind to deletion mutants of tau (ΔPHF6- and ΔPHF6*-2N4R tau) and in mutant forms of tau in which Cys was substituted by Ala (C291, 322A-2N4R and C322A-2N3R tau), we observed associations between ISO, ΔPHF6- and ΔPHF6*-2N4R tau, but not C291, 322A-2N4R and C322A-2N3R tau (Fig. 5e); this demonstrated that ISO binds Cys residues in tau. Based on this and other results reported above, we propose that 1,2-dihydroxybenzene non-ionically binds Cys residues in the MTBR and thus inhibits the formation of tau oligomers by occluding intermolecular tau interactions. We next used mass spectrometry to confirm that 1,2-dihydroxybenzene binds Cys residues in tau; for this, a partial Cys-containing peptide of tau, R3' (skvtskcgslgn; molecular weight (MW)=1,180.3), was analysed after incubation with either the vehicle or the 1,2-dihydroxybenzene-containing compounds, ISO (MW=211.3) and pyrocatechol (MW=110.1). Incubation of the R3' peptide with the vehicle yielded two peaks (*m*/*z*=1,180.5 and 2,358.0), corresponding to peptide monomers and dimers (Fig. 6a); incubation with ISO resulted in a distinct peak (*m*/*z*=1,389.7), as well as peaks corresponding to the R3' peptide monomers and dimers (Fig. 6b and Supplementary Table 1). Peak shifts (*m*/*z*=1,180.6→1,288.5) were also detected after incubation with pyrocatechol (Fig. 6c and Supplementary Table 1), but not with octopamine, a 1-hydroxybenzene containing compound (Fig. 6d). No peak shifts were observed when a mutant Cys→Ala R3' peptide (skvtskagslgn; MW=1,148.2) was incubated with the vehicle (Fig. 6e), ISO (Fig. 6f), pyrocatechol (Fig. 6g) or octopamine (Fig. 6h). Together, these analyses confirmed that ISO could block tau aggregation through interaction of its 1,2-dihydroxybenzene group with Cys residues in tau.

### Effects of ISO in an animal model of tauopathy

**Figure 7:**
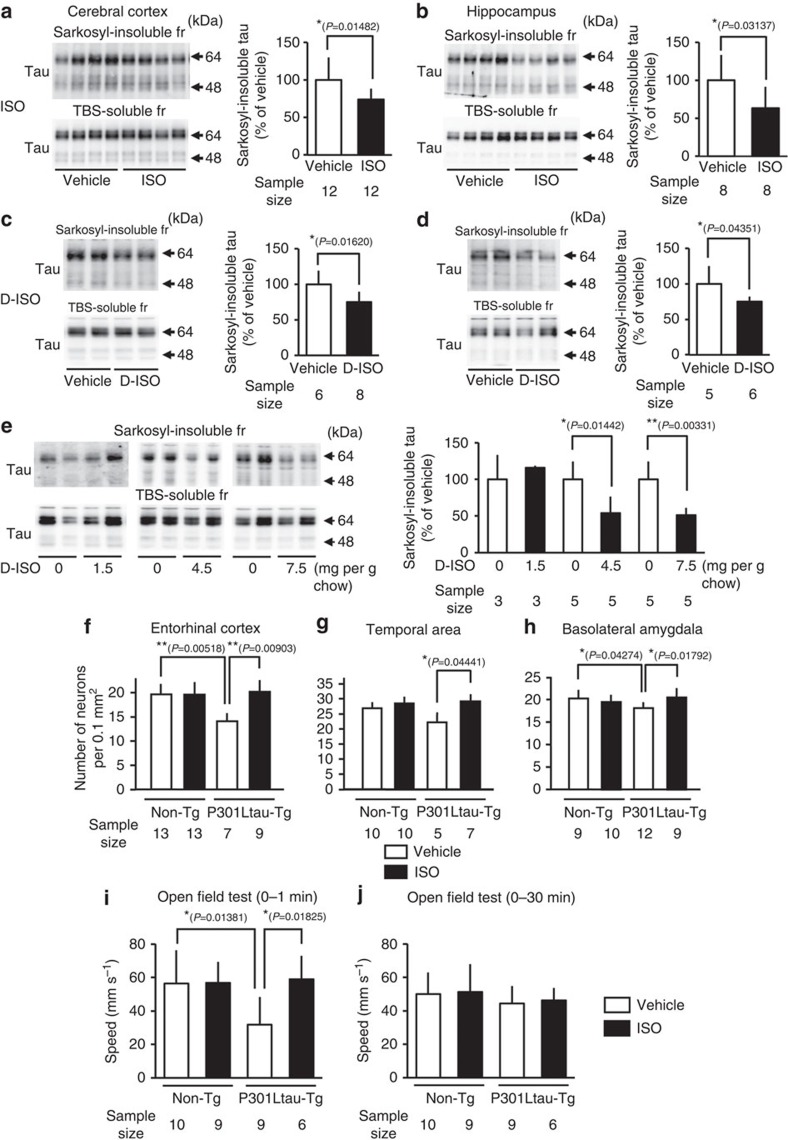
Inhibition of tau aggregation and neuronal loss by isoproterenol (ISO) *in vivo*. Mice expressing human 2N4R tau (P301L) and non-transgenic (Tg) littermate were administrated with vehicle, 1.5 mg ISO (**a**,**b**,**f**-**j**) and D-ISO (**c**,**d**) per g chow for 3 months. The mice were treated with 1.5–7.5 mg D-ISO per g chow for 2 months (**e**). In the cerebral cortex (**a**,**c**) and hippocampus (**b**,**d,e**), levels of Sarkosyl-insoluble and TBS-soluble tau were analysed by western blot and quantified. JM antibody that recognized total tau was used for detection. Densitometry of tau immunoreactivity was quantified and intensity levels of Sarkosyl-insoluble tau normalized to that of TBS-soluble tau. Results are shown as percentage of control (mean±s.d. of 3–12 samples from 3–8 mice from three repeated independent experiments). *P<0.05; **P<0.01 (unpaired Welch's (**a**) and Student's *t*-tests (**b**-**e**)). Coronal sections (4-μm) of mice administrated the vehicle and ISO for 3 months were stained with cresyl violet. The number of neurons in entorhinal cortex (**f**), temporal area (**g**) and basolateral amygdala (**h**) were counted under a microscope assisted with the Neurolucida tracing system. Results are shown as mean±s.d. of 5–13 samples from 3–5 mice from two independent experiments. *P<0.05; **P<0.01 (one-way analysis of variance (ANOVA), with Tukey's multiple comparisons test). Locomotor activity in ISO-treated and untreated mice was monitored in an open field arena (50 × 50 × 40 cm^3^) as described in the Methods. Velocity over the 1st minute (**i**) or 30th minute of testing (**j**) was determined by computing the time intervals between sequential positions of the mice in each video frame. Results are shown as mean±s.d. of 6–10 mice. *P<0.05 (one-way ANOVA, with Tukey's multiple comparisons test). Sample sizes in each experiment are presented in each figure, and mice numbers are described in Supplementary Table 2. fr, fraction.

To investigate the efficacy of ISO in inhibiting tau aggregation and subsequent neuronal loss in a relevant animal model of tauopathy, we used a transgenic mouse line that expresses a highly aggregating form of tau, encoded by the human P301L tau gene. Studies were performed in aged mice (17–18 months) as the amount of Sarkosyl-insoluble tau aggregate in the brain increases with age^26^. Animals (non-transgenic controls and P301L tau transgenic mice) received DL-ISO in their chow at dose of 1.5 mg g^−1^ chow; food intake and body weight did not change over the duration (3 months) of treatment (Supplementary Fig. 5). The oral DL-ISO regimen did not alter TBS-soluble levels of tau, but significantly reduced the load of Sarkosyl-insoluble tau in the cortex (26.1±14.5%; *n*=12), *P*=0.01482 (unpaired Welch's *t*-test), Fig. 7a) and hippocampus (36.7±28.3% (*n*=8), *P*=0.03174 (unpaired Student's *t*-test) Fig. 7b), as compared with mice fed the control diet. Appropriate drug dosage was established in pilot studies in wild-type mice; following administration of ISO at 1.5 mg g^−1^ chow for 2 weeks, their blood and brain levels of ISO were 257 and 40 nM, respectively. Potential mediation of the actions of ISO by β1/2 adrenergic receptors was precluded because similar reductions of Sarkosyl-insoluble tau were observed in both cerebral cortex (Fig. 7c) and hippocampus (Fig. 7d) when P301L tau-transgenic mice were fed for 3 months with the D-isomer of the drug (1.5 mg g^−1^ chow), which lacks adrenergic activity (cf. Fig. 7a,b, respectively). In addition, administration of the inactive isomer of ISO for 2 months resulted in dose-dependent reductions of Sarkosyl-insoluble tau (Fig. 7e). The latter two observations indicate that the ability of D/DL-ISO to inhibit tau aggregation are dose- and time-dependent.

Our previous observations that aged P301L tau-transgenic mice display neuronal loss in the entorhinal cortex, temporal lobe and amygdala^26^ were reproduced in the present study (Fig. 7f–h). Having previously attributed these effects to the increased levels of insoluble Sarkosyl-insoluble (aggregated) tau in the brains of aged P301L tau-transgenic mice^26^, we hypothesized that ISO would promote neuronal survival by reducing tau aggregation. Our hypothesis was supported by the results depicted in Fig. 7f–h, where ISO (1.5 mg g^−1^ chow for 3 months) is seen to prevent age-related reductions in neuron numbers in the entorhinal cortex, temporal lobe and basolateral amygdala of aged P301L tau-transgenic mice; the drug did not influence neuron numbers in any of these areas in non-transgenic mice.

On the basis of above findings, we suggest that inhibition of tau aggregation via ISO-capping of Cys residues of tau prevents the aggregation of toxic tau and therefore, neuronal loss. Such a mechanism is supported by the observation that D-ISO (15 mg g^−1^ chow) reduces Sarkosyl-insoluble tau and blocks neuronal loss in the hippocampus of PS19 mice (a model of tauopathy which also displays severe hippocampal neuronal loss^20^); the latter conclusion is supported by the magnetic resonance imaging data presented in Supplementary Fig. 2. Although the neuronal loss observed in P301L tau-transgenic mice was not accompanied by significantly impaired spatial memory in the Morris water maze test^26^, they displayed significantly reduced locomotor activity during the first minute of placement in an open field arena (Fig. 7i). On the other hand, their locomotor activity over 30 min in the open field arena did not differ from that observed in non-transgenic mice (Fig. 7j); these observations suggest that P301L tau-transgenic mice exhibit emotional disturbance without any change in overall motor activity. Interestingly, ISO alleviated the emotional disturbance in aged P301L tau-transgenic mice (Fig. 7i).

## Discussion

Growing evidence for the key role of aggregates of tau protein in AD and FTDP-17 has spurred efforts to explore pharmacological means to maintain tau in its soluble form and thus, to prevent its aggregation.

ISO was identified as a potential inhibitor of tau aggregation after screening a library of small molecules. Detailed analysis revealed that the drug acts by binding Cys residues in tau that prevent tau oligomerization and formation of insoluble tau aggregates. This property of ISO is attributed to the two phenolic hydroxyl groups in the 1,2-dihydroxybenzene backbone, as other compounds sharing this structure (L-3,4-dihydroxyphenylalanine, dopamine, norepinephrine, epinephrine, and ISO), as well as 1,2-dihydroxybenzene itself, also inhibit tau aggregation, albeit to lesser extents and/or ability to easily traverse the BBB. The pre-requisite role of the 1,2-dihydroxybenzene backbone structure is illustrated by the inability of octopamine and 3-methoxytyramine or 3-methoxy-4-hydroxyphenethylamine, whose chemical backbones include 1-hydroxybenzene, to inhibit tau aggregation. Given that ISO is subject to oxidation, our finding that *o*-quinone (adrenochrome) inhibit tau aggregation as effectively as epinephrine (its non-oxidized parent molecule) is important because it demonstrates that the inhibitory actions of 1,2-dihydroxybenzene-containing compounds on tau aggregation do not depend on their oxidative status. Interestingly, previous work demonstrated nucleophilic covalent attachment of the *o*-quinone in 1,2-dihydroxybenzene compounds to the sulfate group in Cys^42^,^43^; consistent with this finding. It would therefore appear that oxidized 1,2-dihydroxybenzene-containing compounds and their *o*-quinone forms bind covalently to the sulfate group of Cys; this view is plausible because we found that the ISO-bound tau bond cannot be broken eluted by 1 M KCl. The site of chemical reaction between Cys residue of tau and *o*-quinone would be regulated by steric and electronic interactions of these two compounds. Further, MS analysis revealed that complexes comprised of a partial tau R3' peptide and ISO include two phenolic hydroxyl groups, but not *o*-quinone. As shown in the schema in Supplementary Fig. 3, quinone binding to the sulfate group of Cys in tau will be followed by electron transfer to quinone and its back-conversion into ISO (personal communication, M.S. and H.K.)^44^. Oxidation during tau aggregation leads to increased disulfide bonding between Cys residues in tau^45^,^46^; however, substitution of Cys to Ala in tau (C291, 322A-2N4R tau) can induce tau aggregation, suggesting that capping of Cys residues of tau by 1,2-dihydroxybenzene precludes the formation of toxic tau aggregates by hindering disulfide bonding and formation of tau oligomers (Supplementary Fig. 4a,b). The aggregation of wild-type tau was significantly attenuated by both 10 and 100 μM of ISO, whereas that of C291, 322A-2N4R tau was only inhibited by the higher dose of ISO (Supplementary Fig. 4c). Besides showing that ISO binds the Cys residue of tau, this observation indicates that ISO also affects PHF6((306) VQIVYK(311))^41^,^47^ binding, and thus increasing the propensity to form β-sheets and inhibiting the formation of seeds for the aggregation of toxic tau.

It deserves mention that, although NFTs themselves do not exert toxic actions^25^, the degree of dementia correlates well with NFT number and the extent of neuronal loss^13^,^14^. Accordingly, the irreversible brain dysfunction observed in dementia may be ascribed to the neuronal loss that follows the aggregation of toxic tau. Support for this view comes from our finding that ISO reverses emotional disturbances associated with the expression of P301L tau (Fig. 7i) and stimulates neural activity in the prelimbic frontal cortex (Supplementary Fig. 6a); the latter is accompanied by contemporaneous reductions in the levels of Sarkosyl-insoluble tau in the prelimbic cortex (Supplementary Fig. 6b).

In summary, we have demonstrated that ISO, a drug that penetrates the BBB, efficiently inhibits the formation of Sarkosyl-insoluble tau in a mouse line carrying an aggressive mutant form of tau (P301L); the P301L mutation is responsible for FTDP-17, and mice expressing it are very prone to tau aggregation and neuronal death. Our results point to the potential of 1,2-dihydroxybenzene-based compounds to prevent and delay tauopathies such as FTDP-17 and AD by preventing neuronal dysfunction and loss restoring behavioural homeostasis. Using a variety of analytical approaches, we propose that the therapeutic efficacy of 1,2-dihydroxybenzene-containing compounds hinges on their ability to hinder intermolecular interactions between tau molecules by binding to the Cys residues of tau. No untowards reactions to chronic (3 month) ISO exposure were observed in this study; further, oral administration of the pharmaceutical PROTERENOL S (0.25 mg kg^−1^) to cynomologus monkeys for 8 weeks was not accompanied by adverse effects (unpublished observations). Nevertheless, the caveat that toxicity might result from covalent modification of Cys residues in other proteins must be considered during subsequent development of ISO-related compounds for therapeutic use. On the other hand, it should be noted that ISO is commonly prescribed for conditions such as asthma and bradycardia, with the therapeutic dosages in humans being almost fivefold those that we found to be effective for disrupting tau aggregation in mice^48^. The present work suggests a promising novel therapeutic target in the management of tauopathies; its screening strategy and description of a series of compounds that display nucleophilie for Cys residues in tau^49^, provide an important lead in this respect.

## Methods

### Materials

Six-thousand seven-hundred eighty-eight small molecular compounds were obtained from the RIKEN Natural Products Depository (NPDepo) and dissolved in dimethyl sulfoxide to obtain a stock solution (10 mM or 10 mg ml^−1^). The polyclonal anti-total tau antibody (JM) was prepared as previously described^50^. The monoclonal anti-total tau antibody (tau5) was purchased from Invitrogen. All other reagents were of analytical grade and purchased from Nacalai Tesque Inc., Sigma-Aldrich Corp. and WAKO Pure Chemical Industries.

### Chemical array screening

The method is based on identification of associations between a protein of interest with a library of candidate chemicals. In this study, the array consisted of 6,788 small-molecule compounds (RIKEN Natural Products Depository, *NPDepo*) immobilized under ultraviolet radiation on to photo-affinity linker-coated glass slides; the assay was performed using a previously reported method^51^,^52^,^53^ with slight modifications. Briefly, after blocking with 1% skimmed milk in 10 mM HEPES/100 mM NaCl for 1 h at room temperature, the slides were treated with recombinant tau protein in 10 mM HEPES/100 mM NaCl/0.05% Tween for 16 h at 4 °C. The slides were then probed with anti-tau antibody (JM) for 4 h, followed by incubation with a Alexa 633-labelled secondary antibody for 1 h at room temperature. Signal detection was performed with a GenePix 4100A microarray scanner (Molecular Devices), equipped with a 635-nm laser and 655–695 nm band-pass emission filter. When Fraction 1 (soluble tau) was used as bait, 86 out of 6,788 compounds showed potential association with tau. In contrast, none of the compounds tested showed associations either Fraction 3 (granular tau oligomer) or Fraction 6 (fibrilar tau).

### Preparation of recombinant tau protein

Various human tau cDNA was constructed in a pRK172 vector based on the longest form of human wild-type tau encoded 441 amino acid (2N4R); deletion of amino acids at positions 252 to 376 (ΔMTBR), positions 306 to 311 (ΔPHF6), positions 275 to 280 (ΔPHF6*) and with substitutions at C291A and C322A (C291, 322A). A construct that encoded 2N3R tau with a mutation at C322A (2N3R-C322A) was produced. Each recombinant tau was expressed in *Escherichia coli* BL21 (DE3) and purified by modified method reported previously^33^. After *E. coli* expressing tau was sonicated and boiled, recombinant tau proteins in the heat-stable fraction was purified by ion-exchange chromatography (P11; GE Healthcare, or Cellufine Phosphate; JNC Corp.), ammonium sulfate fractionation, gel filtration chromatography (NAP10 column; GE Healthcare) and reverse phase-HPLC (COSMOSIL Protein-R Waters; Nacalai Tesque Inc.). After freeze-drying, recombinant tau proteins were dissolved in milliQ water and stored at −80 °C as a stock solution.

### ThT assay

ThT binding was measured with modified method reported previously^33^. Recombinant wild-type 2N4R tau (10 μM), compounds (indicated concentration) and ThT (10 μM) were mixed in the HEPES buffer (10 mM HEPES, pH=7.4; 100 mM NaCl), and incubated with heparin (0.06 mg ml^−1^; Acros Organics) at 37 °C. At specific time points, fluorescence generated by the binding of ThT to tau aggregates was measured (excitation wavelength: 444 nm; emission wavelength: 485 nm). The tau aggregation mixture was collected 120 h after incubation, and analysed with a pelleting assay, sucrose density gradient centrifugation or AFM.

### Sucrose density gradient centrifugation

Sucrose density gradient centrifugation was performed as described previously^33^. Tau aggregation mixture (1 ml) was layered on top of sucrose step gradients (each 1 ml of 10, 20, 30, 40 and 50% sucrose in HEPES buffer (pH=7.4)) was centrifuged (50,000 r.p.m., 2 h) in a MLS50 rotor (Beckman Coulter) and separated into fractions. Pellet (Pel; Fraction 6) was suspended in 1 ml of buffer containing HEPES buffer, and the recovered tau in each fraction was evaluated with western blotting.

### Pelleting assay

Pelleting assay was performed by modified method reported previously^54^. After ThT assay, samples including tau aggregates were centrifuged (70,000 r.p.m., 2 h) in TLA100.4 or TLA110 rotors (Beckman Coulter) and separated into supernatant and pellet. After the pellet was suspended and sonicated in Laemmli SB including 2-mercaptoethanol, they were boiled for 5 min.

### Cell culture

Neuro2A cell line stably expressing myc-tagged tau (P301L) was established according to a previously described method^38^. Cells were cultured in Dulbecco's Modified Eagle's medium supplemented with 10% fetal bovine serum, 50 μg ml^−1^ gentamicin, 5 μg ml^−1^ puromycin and 1 mg ml^−1^ G418 at 37 °C under 5% CO_2_. Cells at 80–90% confluency were plated in mediums without puromycin and G418. Cells were treated with ISO (0.01, 0.1 and 1 μM), lithium chloride (10 mM) as a positive control that is a glycogen synthase kinase inhibitor, or sodium chloride (10 mM) as a negative control. To confirm the relationship between tau aggregation with β adrenergic effect by ISO, cells were treated with 1 μM ISO, 30 min after pretreating with or without 1 or 10 μM propranolol, which is a competitive adrenergic β blocker. Cells were sonicated in modified RIPA buffer containing 50 mM Tris (pH=7.4), 1% NP-40, 0.25% sodium deoxycholate, 150 mM NaCl, 1 mM ethylene glycol tetraacetic acid (EGTA), protease inhibitors (5 μg ml^−1^ pepstatin, 5 μg ml^−1^ leupeptin, 2 μg ml^−1^ aprotinin and 0.5 mM 4-(2-aminoethyl)benzenesulfonyl fluoride hydrochloride), and phosphatase inhibitors (1 mM okadaic acid, 1 mM Na_3_VO_4_ and 1 mM NaF) 48 h after treatment. The lysates (1 ml) were layered onto 0.32 M sucrose containing 10 mM Tris (pH=7.4), 0.8 M NaCl and 1 mM EGTA, and centrifuged (15,000 r.p.m., 10 min, 4 °C) in an ARO15–24 rotor (Tomy Seiko). The upper part (1 ml) was transferred to 1.5-ml tubes (357448; Beckman Coulter), centrifuged (50,000 r.p.m., 30 min, 4 °C) in a TLA55 rotor (Beckman Coulter) and separated into supernatant (RIPA-soluble fraction) and pellet. After the pellets were sonicated in 1% SDS/RIPA and 1% SDS/TBS buffers, they were centrifuged (50,000 r.p.m., 1 h, 4 °C) in a TLA55 rotor and separated into supernatant and pellet. Pel (SDS-insoluble fraction) was dissolved in 70% formic acid and air-dried. Samples from RIPA-soluble and SDS-insoluble fractions were dissolved in Laemmli SB including 2-mercaptoethanol, and then boiled for 5 min.

### AFM observation

Morphology of the recombinant tau aggregate was observed under AFM^33^. After ThT assay, samples including tau aggregates were loaded to mica and incubated at room temperature for 30 min in a moist box. The mica was washed with milliQ water and then cantilever (OMCL-TR400PSA; Olympus) detected tau aggregate with 3D-Stand Alone AFM (Asylum Research) under the tapping mode. Major and minor axis of tau aggregates and the number of tau aggregates were determined by image analysis using Matlab-based software (MathWorks Co. Ltd.).

### Tau oligomerization assay

After recombinant wild-type 2N4R tau protein (10 μM), and ISO (100 μM) or methylene blue (100 μM) mixing with buffer containing HEPES (10 mM, pH=7.4) and NaCl (100 mM), heparin sulfate (0.06 mg ml^−1^) was added, and incubated at 37 °C. In each time point 10–60 min, tau aggregation mixture were collected and dissolved in Laemmli SB without 2-mercaptoethanol. Tau protein in the samples was separated in SDS–polyacrylamide gel electrophoresis gel without dithiothreitol buffer and visualized in western blotting (Figs 4c and 5).

### Binding assay with FG beads

To determine binding of tau and ISO, we prepared Streptavidin FG beads (5 mg ml^−1^; Tamagawa Seiki) reacted with biotinylated ISO (62.5 μM; Supplementary Fig. 1A), and Streptavidin FG beads (5 mg ml^−1^) reacted with biotin (62.5 μM) as a negative control. Avidin-biotin complexes were formed by incubation for 1 h at 4 °C. After recombinant wild-type 2N4R tau (0.6 μM; input) was reacted with the complexes by rotation (4 h at 4 °C), they were centrifuged and separated into pellet and supernatant. After washing the pellet with 100 mM KCl buffer (pH 7.4) thrice, the pellet was suspended and incubated in 1 M KCl solution for 5 min on ice. The suspension was centrifuged, the supernatant (KCl) was analysed, or the pellet was suspended in Laemmli SB without 2-mercaptoethanol, incubated for 15 min at 60 °C and separated into Pel and supernatant (SB; Supplementary Fig. 1b). The Input, KCl and Pel were solubilized or resuspended in Laemmli SB. Tau protein in the samples was detected with western blotting. To determine binding region, R1 (tapvpmpdlknvkskigstenlkhqpgggk), R2 (vqiinkkldlsnvqskcgskdnikhvpgggs), R3 (vqivykpvdlskvtskcgslgnihhkpgggq) or R4 (vevksekldfkdrvqskigsldnithvpgggn) peptides (120 μM) were pretreated with the avidin-biotin complexes before incubation with recombinant wild-type 2N4R tau.

### MASS spectrometry

Recombinant R3' (skvtskcgslgn) and mutant Cys→ Ala R3' (skvtskagslgn) peptides (10 μM) were incubated with compounds (100 μM) for 5 days at 37 °C. The samples were diluted 20 times with milliQ water and loaded to spot under presence of α-Cyano-4-hydroxycinnamic acid (10 mg ml^−1^). After drying, the samples were analysed using 4800 plus MALDI Tof/Tof analyzer (Applied Biosystems).

### Animals

Transgenic mice expressing 2N4R tau (P301L) with myc- and flag-tag at N- and C-terminal under CaM kinase II promoter was created previously^26^. Male P301L tau-transgenic mice 17- to 18-month old were treated with ISO or D-ISO (1.5–7.5 mg g^−1^ chow) for 2 or 3 months. The numbers of animals used in each experiment are indicating in Supplementary Table 2. All experimental procedures used in this study were approved by the Committee of Animal Experiments at the National Center for Geriatrics and Gerontology.

### Tissue extraction

P301L tau-transgenic mice were anaesthetized and killed after treatment with the compounds; the hippocampus and cerebral cortex were collected. The tissues were homogenized in TBS buffer containing 50 mM Tris (pH=7.4), 150 mM NaCl, 1 mM EGTA, 1 mM EDTA, protease inhibitors and phosphatase inhibitors. The homogenates were centrifuged (23,000 r.p.m., 15 min, 4 °C) in a TLA55 rotor and separated into supernatant (TBS-soluble fraction) and pellet. Pellets were resuspended in 0.32 M sucrose containing 10 mM Tris (pH 7.4), 0.8 M NaCl and 1 mM EGTA and centrifuged (23,000 r.p.m., 15 min, 4 °C) in a TLA55 rotor. Supernatants were collected and treated with 1% Sarkosyl for 1 h at 37 °C. They were centrifuged (60,000 r.p.m., 1 h, 4 °C) in a TLA100.4 or 110 rotors and separated into supernatant and pellet (Sarkosyl-insoluble fraction). Samples from TBS-soluble and Sarkosyl-insoluble fractions were dissolved in Laemmli SB including 2-mercaptoethanol, and then boiled for 5 min.

### Count of neuronal cells

Number of neuronal cells in the brain was measured with modified method reported previously^26^. After P301L tau-transgenic mice were anaesthetized and transcardially perfused with 10% formalin, brains were fixed in 10% formalin solutions for 48 h. Coronal sections (4 μm) were produced from brains embedded by paraffin, and stained with cresyl violet. We counted the number of neuronal cells in the sections using microscope linked to a Neurolucida tracing system (MicroBrightField Inc.).

### Western blotting

Samples dissolved in Laemmli SB were separated by Novex 3–8% Tris-Acetate Gel (binding assay and tau oligomerization assay; Invitrogen) or SuperSep Ace 5–20% gel (other assays; WAKO Pure Chemical Industries) and transferred onto membranes with semi-dry transfer systems (Bio-Rad Laboratory). The membrane was blocked with 5% milk in PBS-T for 1 h at room temperature. They were probed with antibodies overnight at 4 °C. After washing the membrane with PBS-T, blots were incubated with horseradish peroxidase-linked second antibodies and then examined by enhanced chemiluminescence detection on Las3000 or Las4000 (GE Healthcare).

### Open field test

Mice were placed in the centre of an open field apparatus (50 × 50 × 40 cm^3^; O'Hara Co., Ltd.), and their locomotor activity was monitored with a CCD camera; digital data of real-time images were recorded using the public domain NIH Image J software (http://rsb.info.nih.gov/nih-image/). Images were sampled at 2 Hz. Data were analysed using customized Matlab-based software, using an image analysis tool box (Mathworks Co. Ltd.). During testing, the sequential position of the mouse was determined in each video frame from which locomotor speed was calculated.

### Mn-enhanced magnetic resonance imaging

Mn-enhanced MRI was performed as previously described^55^. Mice were given an intraperitoneal injection of 30 mM MnCl_2_ (100 μmol kg^−1^) and returned to their home cages. After 1 h, mice were exposed successively to three different novel places separated by a small (diameter, 30 cm; height, 30 cm) and big (diameter, 60 cm; height, 30 cm) transparent plastic wall and was allowed to explore for 120 min. Thereafter, mice were returned to their home cages for 1 h before being anaesthetized with isoflurane (0.5–1.5% in air) and placement in a Bruker 3T MR scanner; during scanning, breathing and depth of anaesthesia was continuously monitored, with breathing rate maintained at 80–100 breaths per min. Mn-enhanced MRI images were visualized with open-source Osirix software (version 2.5) that allowed navigation through multidimensional DICOM images; relative regional brain activity was determined by measuring MR signal intensities, normalized to the mean signal intensity in the dorsal striatum.

### Measurement of ISO levels in the blood and brain

Male C57BL/6J mice (*n*=7) were administered ISO (1.5 mg g^−1^ chow) for 2 weeks before sacrifice when blood plasma and whole brain were collected. The samples were deproteinized using acetonitrile before determination of ISO concentrations using an LC-MS instrument (UPLC/Quattro Premier XE; Waters).

### Statistical analysis

Data are expressed as means±s.d. The significance of differences between two groups was assessed by Student's or Welch's *t*-tests, and differences between multiple groups were assessed by one-way analysis of variance and Tukey's multiple comparisons test, using PRISM4 (GraphPad Software Inc.). *P*<0.05 was considered statistically significant.

## Additional information

**How to cite this article:** Soeda, Y. *et al.* Toxic tau oligomer formation blocked by capping of cysteine residues with 1,2-dihydroxybenzene groups. *Nat. Commun.* 6:10216 doi: 10.1038/ncomms10216 (2015).

## Supplementary Material

Supplementary Information Supplementary Figures 1-6, Supplementary Tables 1-2 and Supplementary References
