# Supplementary material for: Toxic tau oligomer formation blocked by capping of cysteine residues with 1,2-dihydroxybenzene groups
Source: Nat Commun. 2015 Dec 16;6:10216. doi: 10.1038/ncomms10216 (PMC4703892; doi:10.1038/ncomms10216)
Supplement: Supplementary Information — Supplementary Figures 1-6, Supplementary Tables 1-2 and Supplementary References [file ncomms10216-s1.pdf]

Supplemental Information

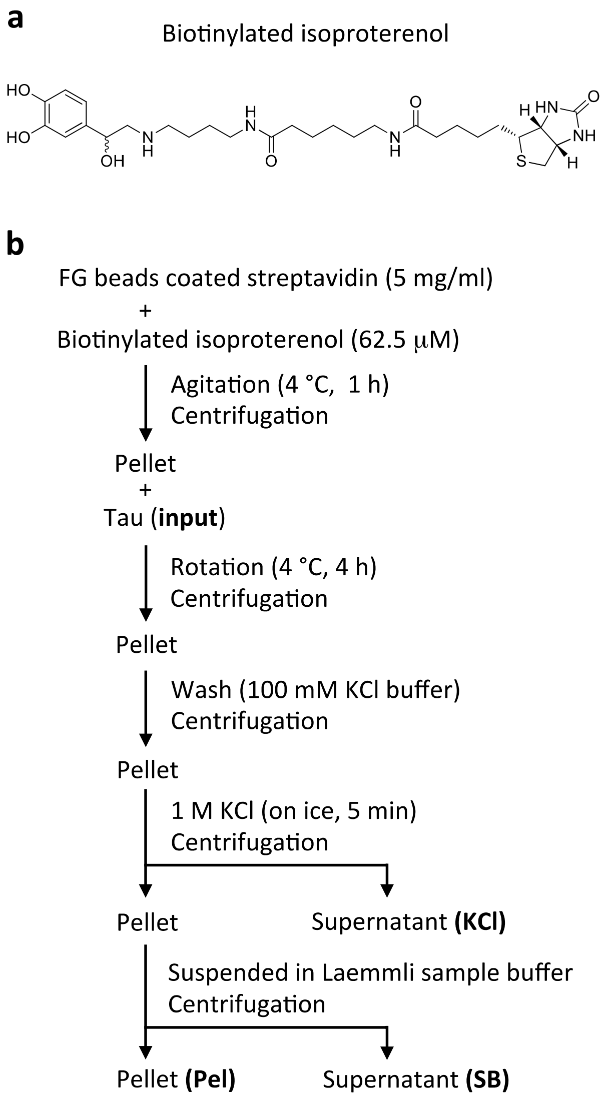

Supplementary Figure 1. Scheme of tau-isoproterenol binding assay. (**a**) Biotinylated isoproterenol was synthesized. (**b**) The binding assay involved the following steps: biotinylated isoproterenol was incubated with streptavidin-coated FG beads (1 h); isoproterenol-coated FG beads were incubated with various tau proteins; the tau-bound isoproterenol was obtained in SB fraction. Biotin/streptavidin-coated FG beads served as control.

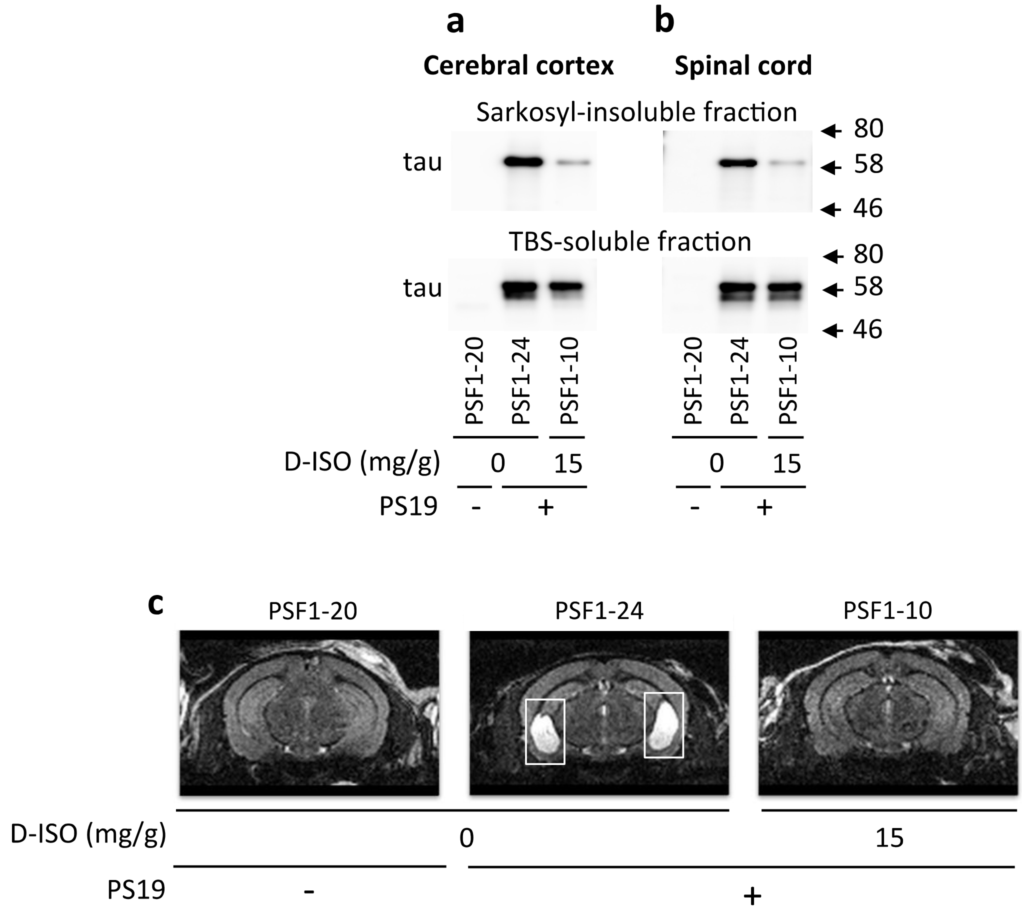

Supplementary Figure 2. Inhibition of tau aggregation and neuronal loss by D-isoproterenol in P301S tau-transgenic (PS19) mice. PS19 mice expressing human 2N4R tau (P301S) were administered D-isoproterenol (D-ISO; 15 mg/g chow) for 11 months, starting at 1 month of age. Sarkosyl-insoluble (upper panel) and TBS-soluble (lower panel) tau in cerebral cortex (**a**) and spinal cord (**b**) of the mice was detected by immunoblotting tissue lysates with JM antibody which recognizes total tau. Structural brain imaging when mice were 12 months old revealed larger cerebral ventricles in close proximity to the hippocampus in PS19 mice that had not received dietary D-ISO (PSF1-24), as compared to non-transgenic control (PSF1-20) and D-ISO-treated PS19 mice (PSF1-10) (**c**). Abbreviations: ISO, isoproterenol.

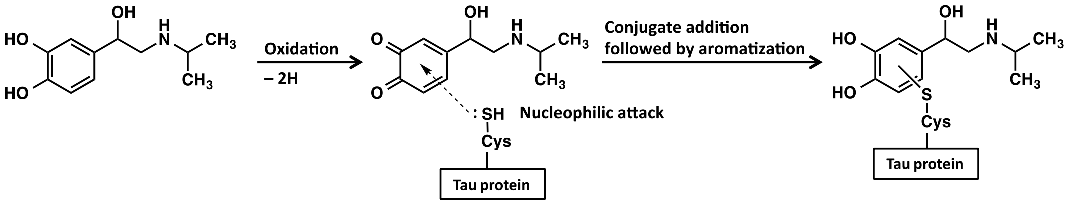

Supplementary Figure 3. Interaction of tau with isoproterenol, a 1,2-dihydroxybenzene-containing compound. Isoproterenol was oxidized to form *o*-quinone. The *o*-quinone part was covalently bound with a sulfur atom at Cys residues on tau via nucleophilic attack (conjugate addition or extended conjugate addition followed by aromatization) [1, 2], and predictedly returned to being a phenolic hydroxyl group.

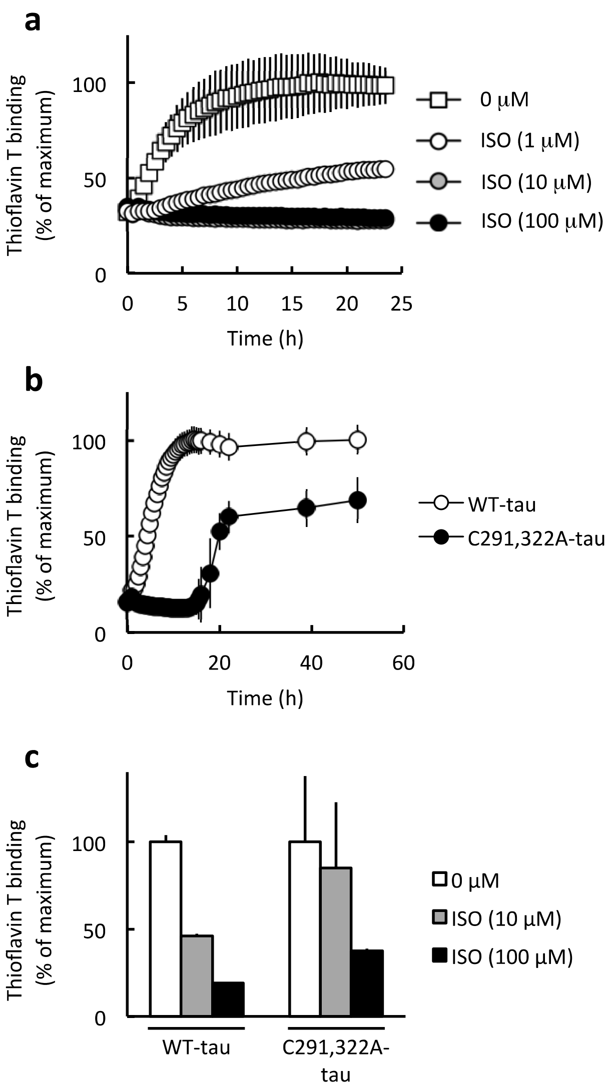

Supplementary Figure 4. Dose-dependency and specificity of inhibition of tau aggregation by pretreatment with isoproterenol, evaluated by the thioflavin T binding assay. (**a**) Tau polymerization was induced by heparin (0.06 mg/ml) in the presence of recombinant wild-type 2N4R tau (10 μM) pretreated with isoproterenol (ISO; 1, 10, and 100 μM) and thioflavin T (10 μM). Note that inhibition of tau aggregation was observed with even just 1 μM of isoproterenol. The vehicle-control was milliQ water. (**b**) Polymerization of C291, 322A-2N4R tau (10 μM) was induced by heparin in the presence of thioflavin T. Thioflavin T binding was measured at the

times indicated. (**c**) Polymerization of wild-type 2N4R tau (10 μM) and C291, 322A-2N4R tau (10 μM) after treatment with ISO (0, 10, 100 μM); polymerization was monitored after 68 h incubation. While tau polymerization was significantly inhibited by more than 10 μM ISO treatment, polymerization of C291, 322A-4R2N tau was only inhibited by 100 μM ISO, suggesting that higher doses of ISO also bind to the PHF6 region. Results are shown as mean ± SD of triplicate (**a**), sextuplicate (**b**) and quadruplicate (**c**) experiments. Abbreviations: ISO, isoproterenol; WT, wild-type.

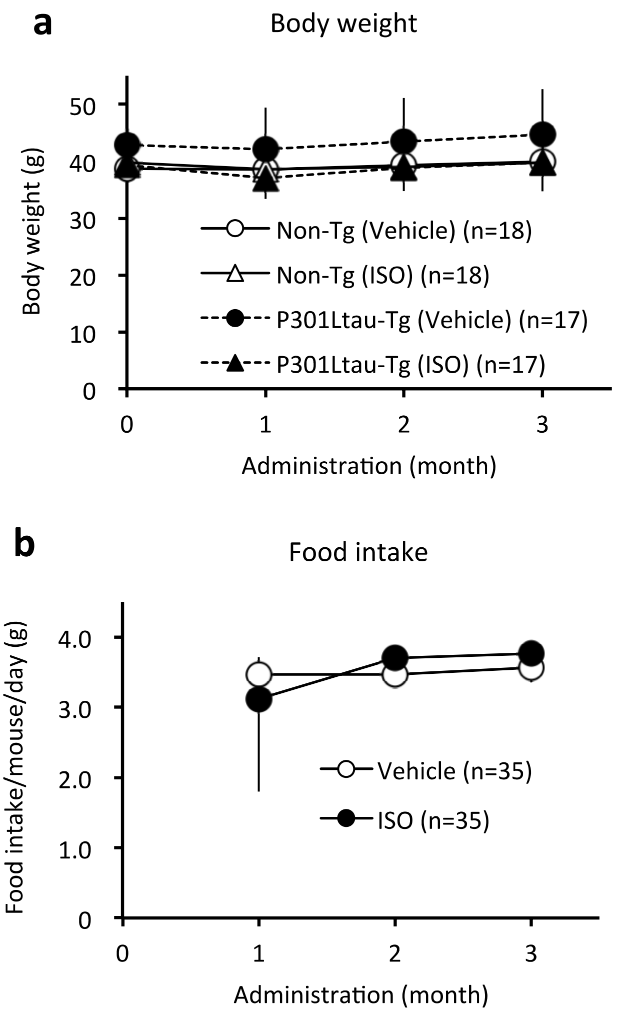

Supplementary Figure 5. Body weights and food intake of P301L tau-transgenic mice during oral administration of isoproterenol. Mice expressing human 2N4R tau (P301L) and non-transgenic littermates received standard chow or chow containing isoproterenol (ISO) at 1.5 mg/g for 3 months. Body weight (**a**) and food intake (**b**) did not differ between vehicle- and ISO-treated mice. Results shown are mean ± SD. Abbreviations: ISO, isoproterenol; Tg, transgenic mice.

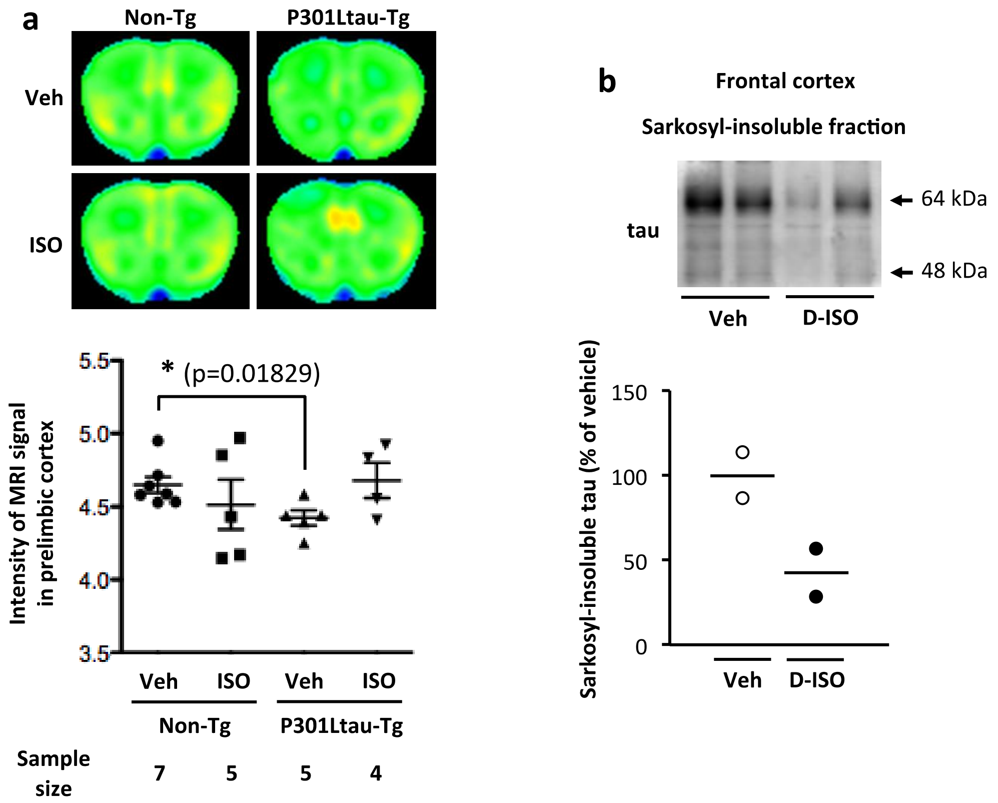

Supplementary Figure 6. Impairment of frontocortical neural activity and increased tau aggregation are reversed by isoproterenol. (**a**) P301Ltau-transgenic mice expressing human 2N4R tau (P301L) and non-transgenic littermates were given isoproterenol (ISO) (1.5 mg/g chow) or vehicle for 3 months. Mn-enhanced MRI (MEMRI) was used to monitor neural activity in the frontal cortex *in vivo*. Intensity of MRI signal, representing neural activity, in the prelimbic cortex was quantified (lower panel). Results are shown as mean ± SE (n = 4–7 mice). * $P < 0.05$ (unpaired Student's *t*-test). (**b**) P301Ltau-transgenic mice were given D-ISO (1.5 mg/g chow) or normal chow for 3 months. Levels of Sarkosyl-insoluble tau in the frontal cortex were analyzed by western blot, using JM antibody which recognizes total tau. Tau immunoreactivity was quantified by densitometry and intensity levels of Sarkosyl-insoluble tau were normalized to levels of total TBS-soluble tau. Results are shown relative to control values. Abbreviations: ISO, isoproterenol; Tg, transgenic mice; Veh, vehicle.

Supplementary Table 1. Expected molecular weights of 1,2-dihydroxybenzene compounds bound to R3' peptide (residue, skvtskcgslgn).

Name	Structure	R1; -OH<br>R2; -OH	R1; =O<br>R2; -OH	R1; =O<br>R2; =O
Isoproterenol	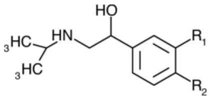	1389.6	1388.6	1387.6
Pyrocatechol	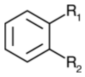	1288.3	1287.3	1286.3

Supplementary Table 2. Summary of number of mice and samples in *in vivo* experiments.

Figure	Mouse	Treatment	Number of	
			Mice	Samples
7a	P301Ltau-Tg	Vehicle	6	12
		1.5 mg ISO/g chow	6	12
7b	P301Ltau-Tg	Vehicle	8	8
		1.5 mg ISO/g chow	8	8
7c	P301Ltau-Tg	Vehicle	3	6
		1.5 mg D-ISO/g chow	4	8
7d	P301Ltau-Tg	Vehicle	5	5
		1.5 mg D-ISO/g chow	6	6
7e	P301Ltau-Tg	Vehicle	3	3
		1.5 mg D-ISO/g chow	3	3
		Vehicle	5	5
		4.5 mg D-ISO/g chow	5	5
		Vehicle	5	5
		7.5 mg D-ISO/g chow	5	5
7f	non-Tg	Vehicle	5	13
		1.5 mg ISO/g chow	5	13
	P301Ltau-Tg	Vehicle	3	7
		1.5 mg ISO/g chow	4	9
7g	non-Tg	Vehicle	5	10
		1.5 mg ISO/g chow	5	10
	P301Ltau-Tg	Vehicle	3	5
		1.5 mg ISO/g chow	4	7
7h	non-Tg	Vehicle	3	9
		1.5 mg ISO/g chow	4	10
	P301Ltau-Tg	Vehicle	4	12
		1.5 mg ISO/g chow	4	9
7i and j	non-Tg	Vehicle	10	
		1.5 mg ISO/g chow	9	
	P301Ltau-Tg	Vehicle	9	
		1.5 mg ISO/g chow	6	

Supplementary References

1 Jagoe, C. T., Kreifels, S. E. & Li, J. Covalent binding of catechols to Src family SH2 domains. *Bioorg. Med. Chem. Lett.* **7**, 113-116 (1997).

2 Lipton, S. A. Pathologically activated therapeutics for neuroprotection. *Nat. Rev. Neurosci.* **8**, 803-808 (2007).
